# Implementation of Mexico’s Health Promotion Operational Model

**Published:** 2008-12-15

**Authors:** Carlos Santos-Burgoa, Lucero Rodríguez-Cabrera, Lilia Rivero, Jorge Ochoa, Adriana Stanford, Ljubica Latinovic, Gretel Rueda

**Affiliations:** Ministry of Health; General Directorate of Health Promotion, Mexico City, Mexico; General Directorate of Health Promotion, Mexico City, Mexico; General Directorate of Health Promotion, Mexico City, Mexico; General Directorate of Health Promotion, Mexico City, Mexico; General Directorate of Health Promotion, Mexico City, Mexico; General Directorate of Health Promotion, Mexico City, Mexico

## Abstract

Mexico is undergoing profound health reform, extending health insurance to previously uninsured populations and changing the way health care services are delivered. Legislation enacted in 2003 and implemented in 2004 mandated funding and infrastructure that will allow 52% of Mexico's population to access medical care at no cost by 2010. This ambitious social reform has not been without challenges, particularly financial sustainability. Health promotion, because of its potential to prevent or delay chronic diseases and injuries and their associated costs, is a key component of health care reform (1).

In 2006, the Ministry of Health's General Directorate of Health Promotion developed the Health Promotion Operational Model. Based on Ottawa Charter functions, the model integrates health promotion activities within the overall health care system. The main goal of this model is to build strong human capital and to improve organizational capacity for health promotion starting at the local level by training health care personnel to implement health promotion activities. Organizational development workshops started in 2006, and implementation plans in all 32 Mexican states were in place by end of 2008 (2).

## Introduction

Before 1917, public health was in the hands of local authorities. The Board of Health of Mexico City had a limited scope, restricted to Federal District. Under the revolutionary principles in the Constitution of 1917, the old Council of Health was transformed into the new Department of General Health (*Departamento de Salubridad General*) with federal jurisdiction and powers to mandate public health measures throughout the country.

The first organized health system to give medical care to the formally employed population and their families was the Mexican Social Security Institute (*Instituto Mexicano del Seguro Social*, or IMSS), founded in January 1943. By October of the same year the Ministry of Health was created and started providing health services to people who did not have access to IMSS, for a minimum fee. Finally, in 1959, the Institute of Security and Social Services for Federal Government Employees was created (1).

Eventually, these 3 institutions began to provide health services to the armed forces and the industry employees, and difficulties started to emerge, such as duplication of functions, inefficiency, and inequity in services (3). Furthermore, because people using these services were considered merely passive recipients of health care, they perceived these institutions as being primarily responsible for their health rather than taking responsibility for their own health.

Health care reform was introduced to address these challenges and the emerging issues of new infectious and noninfectious diseases, mental illness, a rapid increase in violence, and lack of health care access (4). More than half of Mexican citizens lacked access to health services, and out-of-pocket medical expenses could easily consume 30% to 50% of their family monthly income (4,5). This catastrophic spending was impoverishing an estimated 2 to 4 million families every year by 2000 (6).

The following hypothetical example illustrates the situation for many Mexican families before the introduction of health care reform:

Elena lived in a rural area of Veracruz and was diagnosed 2 years previously with type 2 diabetes. Her husband is a farm worker who supports his wife, 5 children, and his parents. Like many rural families in Mexico, he usually earns less than US$300 per month. Medical expenses for a family with a diabetic member can be more than US$100 per month. The family had to sell some belongings to pay for Elena's care. Because the family cannot pay for all her treatment, Elena is inconsistent with glucose self-monitoring and use of insulin and thus is at risk for serious complications (6).

Another factor that prompted reform was the World Health Organization's conceptual framework for evaluating the performance of health systems. Because of its high costs for health care in relation to its gross domestic product per capita, Mexico fared poorly in the evaluation compared with other countries (4,7).

The health care system enacted in 2003 is called the System for Social Protection in Health, and it delivers the local health services collectively known as *Seguro Popular* (Popular Health Insurance). *Seguro Popular* provides universal health insurance, subsidized by the federal and state governments, for the 52% of the Mexican population that is not covered by the other social security systems and is mostly marginalized. The main objective of *Seguro Popular* is to ensure that 50 million Mexicans (5) have full access by 2010 (4). To achieve this objective, at least 2 goals have to be met: financing catastrophic disease costs and strengthening the medical infrastructure in areas of high poverty (5).

To be sustainable, health care reform must address the determinants of health and reduce the burden of chronic diseases and injuries. By law, at least 20% of the financial resources for *Seguro Popular* must be used for health promotion and prevention interventions. By spending resources on preventing and delaying disease and not just curing it, costs will be lower and universal coverage will be financially sustainable (5). This premise is fundamental to the National Strategy on Health Promotion and Prevention for Better Health, launched in February 2007. The National Strategy not only assures effective health services but also provides the necessary elements for individuals and the community to control the determinants of their health. With this move forward, health promotion activities are given priority, and health promotion is understood as a basic public health discipline.

## Health Promotion Operational Model

Health promotion in Mexico lacked the information systems and human resources to deliver health promotion services, according to a study done by the Pan American Health Organization in 2005 (8). To address this problem, the General Directorate of Health Promotion designed and launched Health Promotion Operational Model (*Modelo Operativo de Promoción de la Salud*, or MOPS).

MOPS aims to take advantage of every contact that people have with health services to educate them about their health risk factors and train them to prevent or delay chronic disease. Health care visits are a key opportunity for health promotion interventions.

MOPS contributes a conceptual framework for health promotion activities and then establishes the general guidelines to implement them at the local level, giving priority to the health care services but also focusing on schools, worksites, fields where migrants work, and other sites where promotion is critical. The model establishes the personnel structure that services have to have throughout the chain value, the collaborative communication between them, as well as the professional and technical profile, infrastructure, and responsibilities.

The Ottawa Charter for Health Promotion, established in 1986, identifies 5 core functions: build healthy public policy, create supportive environments, strengthen community actions, develop personal skills, and reorient health services (2). MOPS translates these functions into a health promotion service that integrates 7 components (9):

**1) Management of personal determinants.** With health determinants tools people can identify the determinants that influence their health. This assessment can be developed according to age and sex, and biological, cultural, and employment status. People who know their health determinants profile are in a better position to make healthy choices. Mexico has a strategy called Prevention and Health Promotion at the Stages of Life that sets out the basic actions for preventive care during the life stages from birth until death. Health promotion activities are recommended according to the following age groups and sex:

- Newborn
- Children younger than 5 years
- Children aged 5 to 9 years
- Children aged 10 to 19 years
- Women aged 20 to 59 years
- Men aged 20 to 59 years
- Women and men aged 60 and older

To support this Stages of Life strategy, Mexico uses a system of national health cards. This system is designed to allow people to record on health cards their personal profile and their health promotion activities that they perform at each stage of life. Ideally, this card system will encourage the population to improve habits, customs, attitudes, and practices to protect and preserve their health throughout all stages of life.

**2) Health capacity building and competence development.** This component promotes educating the population on values, attitudes, and skills to help them preserve their health and is built through individual health counseling and community workshops.

**3) Social participation for community action.** This component drives organized and informed participation of the population and promotes the creation of social networks. The implementation of health promotion strategies from the social base has community empowerment as a main goal.

**4) Development of healthy environments.** This component advocates the creation of physical and social spaces where people live, study, and work in hygienic, safe, and stimulating conditions to maintain health and improve quality of life. It promotes the protection and conservation of natural resources and the proper management of physical, chemical, and biological agents.

**5) Advocacy.** This component refers to working both within and outside the health sector to promote cooperation with other sectors. Such efforts generate synergy between the various actors, fields, and levels that are or could be involved in providing health promotion services. Advocacy takes place at all levels of government, public and private health institutions, other public institutions, businesses, and nongovernmental organizations.

**6) Social** **marketing in health.** This component seeks to promote healthy attitudes and behaviors. It involves the analysis, planning, execution, and evaluation of programs designed to influence the voluntary conduct of target audiences to improve their personal welfare and that of society. Social marketing uses a mixture of classical marketing techniques — product, place, price, and promotion — mixed with elements such as bonds to finance public and political alliances.

**7) Evidence in health promotion.** Evidence refers to evaluation data on the effectiveness of health promotion interventions and other information that generates knowledge for decision making. The purpose of this component is to collect such data at the local, municipal, state, or national levels, for local decision making.

The following is an example of how the components of MOPS could work together for health promotion:

Fernando, 48, the father of 5 children, works for a computer company in Monterrey, Nuevo León. He gets up early every day, almost never eats breakfast, sits all day in front of the computer, and does not practice any kind of physical activity. At lunch, he frequently consumes fast food (tacos, fried food, and sweet bread) with soda.

Six months ago, he took his daughter Alejandra to a health center in response to an immunization campaign. The nurse who attended the child took the opportunity to present Fernando the National Health Card for men aged 20 to 59 years (which lists preventive screening recommendations) and performed a basic checkup. She created a personal risk profile for him and recommended that he attend an education program on healthy food and physical activity.

Fernando started exercising every afternoon after work, eating more nutritious meals, and even motivated his wife and son to join him.

Some weeks later, while walking with his wife, they were almost hit by a speeding truck on a street close to the community playground. Fernando organized his neighbors into a group that proposed an initiative for safer and healthier public spaces to local authorities. The authorities acknowledged the importance of the request, and together with some local companies started to create healthier environments by reorganizing public areas.

Fernando's community improved. Because of the MOPS health marketing component, many people learned about these changes. Surveys collected evidence of the effectiveness of these activities.

The MOPS implementation workshops represented a challenge because of the government structure, economic status, and cultural complexity of states. During the workshops, we focus on building the common language of health promotion throughout the managerial system at the states, including directors, program coordinators, and district health officers. The second part of the workshops analyzes the state conditions and develops a local and state light implementation plan, which is ultimately approved by the State Health Secretary. More than 2,000 state and local officers have participated in the workshops.

The workshops motivated state public health authorities to embrace MOPS and work to implement it at all levels. A strong integrated health promotion service is seen as a basic necessity for the National Strategy on Health Promotion and Prevention for Better Health to function, and MOPS plays the main role in that.

## Conclusion

MOPS responds to the growing process of health care democratization and the challenge to sustain it. It demands trained professionals and evidence-based interventions; it is based on local experience but nevertheless is adaptable to all health system levels. MOPS can enable health care reform to be sustainable socially and financially.

MOPS is a new tool that is has made it through a critical phase: the implementation process in each state of Mexico; this is a complex task that requires close monitoring by the state officers and the General Directorate of Health Promotion, which will advise and strengthen states' efforts to implement MOPS. Along the way, the relevance of the model will have to be evaluated and adjusted to the local and national changing realities. This is the next stage.
